# FAMILY PREPAREDNESS AT HOSPITAL DISCHARGE OF THE PERSON LIVING WITH DEMENTIA

**DOI:** 10.1093/geroni/igac059.381

**Published:** 2022-12-20

**Authors:** Marie Boltz, Irene Best, Ashley Kuzmik

**Affiliations:** Penn State, Pennsylvania State University, Pennsylvania, United States; Pennsylvania State University, University Park, Pennsylvania, United States; Penn State University, University Park, Pennsylvania, United States

## Abstract

Family care partners face increased and more complex caregiving demands at the time of hospital discharge for persons living with dementia. This study examined family care partners’ needs for preparation and factors associated with the degree of preparation for caregiving. Care partners who were younger (t=3.26, p=.001), demonstrated anxiety (t= 2.6, p=.010) and depression (t =4.6, p <.001), and were caring for a person in pain (t=2.3, p=.023) had lower scores on the Preparation for Caregiving Scale. Care partners described the least preparation to care for the patients’ emotional needs (M= 2.8, SD=1.2) and deal with the stress of caregiving (M= 2.7, SD=1.2). Content analysis of nurses’ transitional care notes converged with these findings, while also describing a lack of preparation to advocate for information from medical providers. Findings suggest the need to attend to the psychological and informational needs of care partners, while considering patient well-being and comfort.

